# Progress towards the elimination of vertical transmission of HIV, syphilis and hepatitis B in 21 high‐burden countries

**DOI:** 10.1002/jia2.70091

**Published:** 2026-03-26

**Authors:** Melanie M. Taylor, Rania A. Tohme, Robert McDonald, Michele Montandon

**Affiliations:** ^1^ Division of HIV Prevention U.S. Centers for Disease Control and Prevention Atlanta Georgia USA; ^2^ Division of Viral Hepatitis U.S. Centers for Disease Control and Prevention Atlanta Georgia USA; ^3^ Division of STD Prevention U.S. Centers for Disease Control and Prevention Atlanta Georgia USA; ^4^ Division of Global HIV and Tuberculosis U.S. Centers for Disease Control and Prevention Atlanta Georgia USA

**Keywords:** antenatal care, hepatitis B, HIV, prevention, syphilis, treatment

## Abstract

**Introduction:**

Elimination of mother‐to‐child transmission (EMTCT) of HIV, syphilis and hepatitis B by 2030 is a global goal endorsed by countries. To achieve EMTCT, countries need to meet targets for programme and impact indicators.

**Methods:**

We examined progress and gaps in service delivery across the three infections among 21 countries with a high burden of HIV, syphilis and/or hepatitis B. We summarized national coverage of interventions to prevent vertical transmission of HIV, hepatitis B and syphilis. Service coverage indicators were extracted from public data sources for the most recent available years (2014−2024).

**Results:**

Antenatal care (ANC) coverage (at least one visit) ranged from 62% to 99%; 14 countries (67%) had coverage ≥90%. Twelve countries (57%) had ANC HIV testing coverage above 90%; 13 countries (62%) reported antiretroviral treatment coverage ≥90%. Eight countries (38%) reported ANC syphilis testing coverage ≥90%; ten countries (48%) reported ≥90% treatment coverage among pregnant women diagnosed with syphilis. All 21 countries have introduced three infant doses of hepatitis B vaccine (HepB3), and six countries have introduced the hepatitis B birth dose vaccine (HepB‐BD) in their national immunization schedule. Among the six countries that provide HepB‐BD, only four reported coverage with timely HepB‐BD given within 24 hours of birth (range 52–70%). Seven (33%) countries have achieved HepB3 coverage ≥ 90%.

**Discussion:**

Antenatal service delivery for prevention of mother‐to‐child transmission of syphilis and hepatitis B lags behind that of HIV. Despite the availability of ANC platforms for the delivery of HIV, syphilis and hepatitis B services, many countries face new and ongoing implementation and funding challenges in reaching global targets for EMTCT of these infections.

**Conclusions:**

Integrating HIV, syphilis and hepatitis B prevention of vertical transmission services into ANC and maintaining support for EMTCT efforts is critical to close these gaps.

## 1 INTRODUCTION

The World Health Organization (WHO) launched its process for country‐level validation of elimination of mother‐to‐child transmission (EMTCT) of HIV and syphilis in 2014. In 2017, WHO added a Path to Elimination (PTE) certification process for high‐burden countries with maternal HIV prevalence ≥2% and maternal syphilis prevalence >1%. EMTCT validation and PTE were expanded to include hepatitis B in 2021, with high burden defined as hepatitis B surface Antigen (HBsAg) prevalence >1% among ≤5‐year‐olds or general population prevalence >5% [1, 2]. For each infection, both process and impact targets must be met for countries to be validated for EMTCT or PTE for at least one of these infections [1]. EMTCT process indicators include coverage of first antenatal care visit (ANC1) (≥95%), testing and treatment of pregnant women for HIV, testing and treatment of pregnant women for syphilis (≥95%), and timely (within 24 hours of birth) hepatitis B birth dose vaccine (HepB‐BD) and three infant doses of hepatitis B vaccine (HepB3) (≥90%) [1]. While infant hepatitis B vaccination is the primary intervention to prevent vertical transmission of the hepatitis B virus (HBV), the WHO recommends hepatitis B surface antigen (HBsAg) testing of all pregnant women and treatment for those diagnosed with hepatitis B [2]. EMTCT impact targets include the population case rate of new paediatric HIV infections due to MTCT (≤ 50 infant cases/100,000 live births [LB]), MTCT rate of HIV (<2% in non‐breastfeeding populations or <5% in breastfeeding populations), case rate of congenital syphilis (CS) (≤ 50 per 100,000 LB) and prevalence of HBsAg in children (≤0.1%). PTE impact targets vary by tier (Gold, Silver and Bronze) and include HIV MTCT rates, infant case rates for HIV and syphilis, coverage with HepB‐BD and HepB3 vaccine coverage [1].

Over the past two decades, international donors, national governments and philanthropic organizations have made substantial investments in HIV programmes to prevent MTCT (PMTCT), largely in sub‐Saharan Africa, notably through the U.S. President's Emergency Plan for AIDS Relief (PEPFAR) [3] and the Global Fund to Fight AIDS, Tuberculosis and Malaria [4]. Globally, the number of infants infected with HIV has declined, from over 300,000 in 2010 to 120,000 in 2024, though the rate of decline has slowed in recent years [5, 6, 7]. In 2024, an estimated 240,000 infant HIV infections were averted due to PMTCT interventions. In 2022, an estimated 700,000 infants were born globally with CS [8]. Hepatitis B vaccination has been estimated to have prevented 38 million deaths over the lifetime of persons born during 2000–2030 [9]. In 2022, the global prevalence of chronic HBV infection in children was estimated to be 0.7%, corresponding to 5.6 million children having hepatitis B globally, primarily due to low vaccination coverage in sub‐Saharan Africa [10]. We reviewed progress towards EMTCT of HIV, syphilis and hepatitis B in 21 high‐burden countries that received U.S. PEPFAR support for PMTCT of HIV, to identify programme successes and gaps and highlight potential opportunities to build on the HIV PMTCT and ANC platforms to accelerate prevention, screening and treatment service deliveries for all three infections.

## 2 METHODS

### 2.1 Data and measurement

Twenty‐one countries were included in this review: Angola, Botswana, Cameroon, Cote d'Ivoire, Democratic Republic of Congo (DRC), Eswatini, Ethiopia, Haiti, Kenya, Lesotho, Malawi, Mozambique, Namibia, Nigeria, Rwanda, South Africa, South Sudan, Tanzania, Uganda, Zambia and Zimbabwe.

### 2.2 Data sources

Coverage of ANC as at least one visit (ANC1 coverage), and the percentage of births assisted by a skilled birth attendant (facility delivery coverage) were extracted from the UNICEF data warehouse [11]. ANC1 coverage is defined as the percentage of women aged 15−49 years with a live birth who received ANC care by skilled personnel at least once during pregnancy; ANC1 coverage estimates come from nationally representative household survey data (Table S1).

HIV antenatal testing coverage, antiretroviral treatment (ART) coverage in pregnant women living with HIV, and the MTCT rate for HIV reflect country‐reported and modelled data reported into the Global AIDS Monitoring (GAM) system and reported within publicly available UNAIDS epidemiology reports [7, 12, 13, 14, 15].

Syphilis testing and treatment coverage reflect facility‐based coverage of services in ANC. The numerators are women tested and treated for syphilis in ANC. For syphilis testing, the denominators are all women attending ANC. For treatment of syphilis, the denominators are all women attending ANC who tested positive for syphilis [1]. Most recent data on antenatal syphilis testing and treatment were extracted from country‐reported data within the WHO Global Health Observatory (GHO), as were the impact indicators of reported infant case rates of HIV and syphilis and prevalence of vertical transmission of HIV [12, 16].

WHO/UNICEF estimates for national immunization coverage were used to compile country‐specific coverage estimated for the timely (within 24 hours of birth) hepatitis B birth dose vaccine (HepB‐BD) and three doses of hepatitis B vaccine (HepB3) completed by 6 months of age [17]. Prevalence of HBsAg in children and pregnant women (or women aged 15–49 years) in each country was compiled by review of the latest published data from nationwide serological surveys, including population‐based HIV impact assessment surveys [18, 19, 20, 21, 22, 23, 24, 25, 26, 27, 28, 29]. When national serosurvey estimates were not available, we relied on published modelled estimates of HBsAg prevalence among children or systematic reviews and meta‐analyses for HBsAg prevalence in pregnant women [10, 30].

Country‐reported, publicly available data collected by WHO and UNAIDS were referenced to collect information on national policies and plans for EMTCT of syphilis and hepatitis B in place as of 2023 (Table S1) [31, 32].

## 3 RESULTS

Coverage of services for PMTCT of HIV, syphilis and hepatitis B are presented by country using data from 2024 or the most recent available year (2014−2024) (Table 1).

**TABLE 1:** Country‐reported monitoring indicators for prevention of mother‐to‐child transmission of HIV, syphilis and hepatitis B in 21 countries

	Antenatal care (ANC) and delivery^1^		Disease burden (%) in pregnant women (PW)			HIV indicators			Syphilis indicators^3^			Hepatitis B indicators		
Country	ANC1 coverage (%)	Facility delivery coverage (%)	HIV prevalence (%) in women 15–49 years old^2^	Syphilis test positivity (%) among PW in ANC (year)^b^, ^3^	Prevalence of chronic hepatitis B virus infection in women 15–49 years old or PW % (95% CI)^4^	HIV testing coverage (%) among PW in ANC^2^	Population‐level ART coverage (%) among PW^2^	HIV MTCT rate^2^ (%)	Syphilis testing coverage (%) in ANC (year)	Syphilis treatment coverage in PW (%) (year)	Congenital syphilis case rate per 100,000 live births^c^	Timely Hep B BD vaccination coverage 2024 (%)^[Link]^, ^5^	Hep B3 vaccination coverage 2024 (%)	Prevalence of chronic hepatitis B virus infection in children % (95%CI)^6^
Angola	82	46	2.3	2.54 (2019)	8.7 (6.9−10.6)	94	79	17	15.3 (2019)	88.8 (2019)	NR	NR	64	3.3 (2.5−3.3)
Botswana	94^a^	100	20.5	0.44 (2024)	n/a	86	>98	1	54.8 (2024)	53.7 (2024)	529.4 (2024)	58	95	n/a
Cameroon	89	67	3.3	5.74 (2024)	6.0 (NR)	63	59	15	65.5 (2024)	99.0 (2024)	NR	No HepB‐BD	77	1.6 (1.4−1.6)
Cote d'Ivoire	95	81	2.4	4.87 (2017)	n/a	89	88	9	88 (2017)	NR	410.9 (2018)	70	77	0.8 (0.4−0.8)
Democratic Republic of Congo	82	82	0.9	7.78 (2022)	2.1 (NR)	36	32	31	23.0 (2024)	12.9 (2024)	415.0 (2024)	No HepB‐BD	65	1.2 (1.1−1.3)
Eswatini	99	93	28.1	1.96 (2023)	NR	96	>98	3	48.0 (2023)	30.2 (2023)	NR	No HepB‐BD	84	1.4 (1.2−1.5)
Ethiopia	74	48	0.9	0.82 (2024)	5.4 (3.9−6.9)	98	92	9	78.9 (2024)	94.7 (2024)	5.48 (2024)	No HepB‐BD	73	0.7 (0.6−0.7)
Haiti	91	39	2.1	2.01 (2024)	2.5 (1.7−3.4)	85	60	20	71.3 (2024)	95.0 (2024)	76.2 (2023)	No HepB‐BD	60	0.5 (0.2−1.2)
Kenya	98	88	4.1	0.95 (2023)	2.7 (NR)	95	90	9	93.6 (2023)	44.3 (2023)	NR	No HepB‐BD	91	0.8 (0.7−1.3)
Lesotho	91	89	21.8	3.21 (2024)	n/a	92	94	5	81.5 (2024)	59.6 (2024)	NR	No HepB‐BD	86	1.7 (1.4−1.8)
Malawi	97	97	8.3	2.40 (2024)	n/a	98	96	7	84.8 (2024)	100 (2020)	NR	No HepB‐BD	91	0.4 (0.4−0.5)
Mozambique	87	65	14.5	2.31 (2024)	n/a	98	82	12	99.1 (2024)	97.2 (2024)	417.5 (2014)	No HepB‐BD	70	0.9 (0.7−1.0)
Namibia	97^a^	87^a^	11.7	2.97 (2024)	n/a	98	97	4	98.5 (2024)	NR	296.2 (2024)	67	74	n/a
Nigeria	70	49	1.5	0.15 (2024)	6.1 (5.1−7.0)	81	NR	NR	57.9 (2024)	100 (2024)	NR	52	67	4.5 (3.6−5.6)
Rwanda	98	93	2.6	0.57 (2024)	1.2 (NR)	98	>98	6	94.7 (2024)	NR	131.11 (2024)	No HepB‐BD	98	0.3 (0.2−0.4)
South Africa	94	96	23.0	NR	7.2 (6.7−7.6)	87	>98	3	83.9 (2024)	75.9 (2024)	86.2 (2022)	No HepB‐BD	74	1.1 (1.0−1.2)
South Sudan	62^a^	12^a^	1.9	4.63 (2024)	n/a	79	81	20	98.8 (2024)	100 (2024)	NR	No HepB‐BD	73	2.9 (2.7−3.2)
Tanzania	90	81	4.7	0.40 (2024)	2.8 (NR)	93	>98	8	83.5 (2024)	92.8 (2024)	415.9 (2024)	No HepB‐BD	83	0.4 (0.3−0.5)
Uganda	99	86	6.4	1.35 (2024)	3.1 (NR)	98	>98	6	95.1 (2024)	88.7 (2024)	863.4 (2020)	NR	91	0.3 (0.3−0.4)
Zambia	97	84	12.3	5.95 (2024)	4.1 (NR)	97	98	6	93.1 (2024)	100 (2024)	377.3 (2024)	No HepB‐BD	91	0.4 (0.3−0.6)
Zimbabwe	93	86	12.1	1.78 (2024)	n/a	82	93	6	95.3 (2024)	92.9 (2024)	143.7 (2024)	No HepB‐BD	91	1.1 (1.0−1.3)

***Notes*: ANC1 coverage** is the percentage of women aged 15−49 with a live birth who received ANC care by skilled health personnel at least once during pregnancy. ANC1 coverage estimates come from nationally representative household survey data, such as demographic health surveys and multiple indicator cluster surveys.

**Facility delivery coverage** reflects the percentage of births assisted by a skilled birth attendant; data on institutional births were used for countries in which data on skilled attendance at birth were not available. Facility delivery coverage is based on national household survey data and routine health systems.

Abbreviations: n/a, not available; NR, not reported. ANC: antenatal care, HepB‐BD: birth dose of hepattis B vaccine, HepB3: three doses of hepatitis B vaccine, MTCT: mother‐to‐child transmission, PW: pregnant women.

^a^ Denotes data prior to 2014.

^b^ Syphilis test positivity reflects the use of various screening algorithms that include the use of single tests (non‐treponemal or treponemal) with or without reflex confirmatory testing using non‐treponemal or treponemal tests. These data are not adjusted for test type, and thus, test positivity should not be interpreted as prevalence.

^c^Country‐reported congenital syphilis (CS) case rates reflect national CS case definitions, which may not align with the WHO case definition. Reported CS case rates do not reflect WHO case estimation methods.

^d^Timely HepB‐BD: hepatitis B birth dose vaccine given within 24 hours of birth.

***Data sources***:

^1^ UNICEF data warehouse: https://data.unicef.org/dv_index/ [Accessed 19 Mar 2026].

^2^ UNAIDS epidemiological estimates: aidsinfo.unaids.org. When the 2024 estimate is not available, 2023 or the most recent prior year was used [Accessed 19 Mar 2026].

^3^ Global AIDS Monitoring (GAM) System; WHO Global Health Observatory (GHO) (data from 2017–2024). https://www.who.int/data/gho/data/indicators. Accessed 19 March 2026.

^4^ Reported prevalence estimates are the most recent available data from serosurveys in each country, 2013–2023. Sources are References: [18, 19, 20, 21, 22, 23, 24, 26, 27, 28, 29, 30].

^5^ WHO/UNICEF Immunization estimates: https://immunizationdata.who.int/global/wiise‐detail‐page/hepatitis‐b‐vaccination‐coverage.

^6^ Reported prevalence estimates are the most recent available data from serosurveys or modelled estimates in each country, 2013–2023. Sources are References: [10, 25, 28, 29].

### 3.1 ANC coverage and health facility delivery

ANC1 coverage ranged from 62% to 99% (median 93.0%). Among 21 countries, 14 (66.7%) had ANC coverage of ≥90%, and eight (38.1%) had ANC coverage of ≥95%. Reported ANC1 coverage data was greater than 10 years old in three countries (14.2%). Health facility delivery coverage ranged from 12% to 100% (median 81%) and was ≥90% in five of 21 countries (23.8%) (Table 1).

### 3.2 Maternal HIV, syphilis and hepatitis B prevalence

HIV prevalence in women ages 15–49 as a proxy for maternal HIV prevalence ranged from 0.9% to 28.1% (median 4.7%). Seventeen of the 21 countries (81.0%) are considered to have a high HIV burden (≥2% prevalence). Syphilis test positivity in pregnant women ranged from 0.2% to 7.8% (median 2.2%). Based on syphilis test positivity in ANC, 15 countries (71.4%) are high burden (≥1% maternal prevalence) for syphilis. Of the 12 countries with available data on prevalence of HBsAg among either pregnant women or women aged 15–49 years, prevalence of HBsAg ranged from 1.2% to 8.7%, with 11 (52.4%) countries having intermediate or high endemicity for hepatitis B (HBsAg ≥2%) (Table 1).

### 3.3 Coverage of maternal testing and treatment for HIV and syphilis, and infant hepatitis B vaccination

HIV testing coverage among pregnant women in ANC across the 21 countries ranged from 36% to 98% (median 93%). Twelve countries (57%) had ≥ 90%, and nine countries (42.9%) had ≥ 95% HIV testing coverage. Estimated population‐level ART coverage among pregnant women ranged from 32% to >98% (median 89%). Overall, 13 countries (61.9%) had ≥ 90% and nine countries (42.9%) had ≥ 95% ART coverage among pregnant women (Table 1).

Country‐reported antenatal testing coverage for syphilis ranged from 15% to 99% (median 83.9%). Eight countries reported ≥90% and five (9.5%) reported ≥95% ANC syphilis screening coverage. Treatment coverage of pregnant women with positive syphilis test results ranged from 15% to 100% (median 92.9%). Ten countries reported ≥90% and eight countries reported ≥95% syphilis treatment coverage among pregnant women with positive syphilis tests. Three countries did not report syphilis treatment coverage (Table 1).

Of these 21 countries, 15 (71.4%) have not introduced HepB‐BD in their national immunization schedule. Among the six countries that provide HepB‐BD, only four reported timely HepB‐BD with a coverage ranging from 52% to 70%. All countries have introduced three doses of hepatitis B vaccine given through a combination vaccine at 6, 10 and 14 weeks of age, and coverage ranged from 60% to 98% (median 77%). In 2024, seven (33%) countries have achieved a HepB3 coverage ≥ 90% (Table 1).

### 3.4 HIV MTCT rates, CS case rates and infant HBsAg prevalence

HIV MTCT rate estimates ranged from 1% to 31% (median 7.5%); one country (4.8%) had a MTCT rate ≤2%, and five countries (24%) had MTCT rates ≤ 5%.

CS case rates were reported by 13 (61.9%) of 21 countries and ranged from 5.5 to 863/100,000 LB (median 377.3/100,000 LB). Twelve of thirteen reporting countries (92.3%) reported CS case rates of ≤750 cases per 100,000 LB; one of which reported a case rate ≤50/100,000 LB (Table 1).

Of the 19 countries with available data, the estimated prevalence of HBsAg in children ranged from 0.3% to 4.5% (Table 1). Six of 19 countries (31.6%) had a prevalence of HBsAg ≤0.5% (WHO 2025 target), and 10 (52.6%) countries had a prevalence of HBsAg ≤ 1% (WHO 2020 target).

### 3.5 National EMTCT policies

Of the 21 countries, 18 have national policies for triple EMTCT of HIV, syphilis and hepatitis B. Nineteen countries (90%) have a national plan for PMTCT of syphilis. Eighteen countries (86%) have specific policies for hepatitis B testing in pregnant women.

## 4 DISCUSSION

This country data review demonstrates the wide‐ranging coverage of essential services to prevent the vertical transmission of HIV, syphilis and hepatitis B across 21 high‐burden countries that received PEPFAR support for PMTCT of HIV. Local government investment and leadership is essential to progress towards the EMTCT of HIV, syphilis and hepatitis B. As examples, Botswana was the first country with a high HIV prevalence to have applied for and achieved the WHO Silver tier on the PTE in 2021 [33], followed by the PTE Gold for MTCT of HIV in 2025 [34]. Similarly, in 2024, Namibia achieved the WHO Silver tier and Bronze tier on the PTE of MTCT of HBV and HIV, respectively [35]. Achievements in both countries were a result of strong leadership, well‐developed national policies and plans, dedicated resources and commitment to PMTCT programmes, and a coordinated response across multiple sectors, including maternal and child health, HIV, viral hepatitis and immunization programmes.

Nationally reported coverage of ANC and specific services, primarily maternal screening and treatment, to prevent vertical transmission of these three infections vary among countries in this review. While several have achieved EMTCT‐level (95%) or PTE‐level (90%) coverage for service delivery indicators, others report <50% coverage. Facility delivery coverage was also variable among countries, and though it is not a criterion for EMTCT or PTE validation, key EMTCT interventions may be linked to delivery at the health facility, in particular provision of a timely HepB‐BD [36]. Improving access and uptake of ANC and facility delivery and reaching pregnant women who do not come to ANC or a health facility can improve coverage of key HIV, syphilis and hepatitis B EMTCT interventions.

Whereas programmatic data on PMTCT of HIV are consistently reported within long‐established HIV PMTCT monitoring and evaluation frameworks, data on syphilis and hepatitis B testing and treatment, diagnosis and reporting of CS, and recent national prevalence estimates of hepatitis B in pregnant women and children are less available. As evidenced by the data presented here, many countries do not report CS case counts into global monitoring platforms. In addition, there are no country data on screening and treatment for hepatitis B among pregnant women, limiting our inability to present this information. Opportunities for improvement and integration of syphilis and hepatitis B programmatic data (testing and treatment coverage) into existing HIV PMTCT electronic databases and nationally representative surveys for HIV would inform local programming for service delivery and monitoring of national indicators for triple EMTCT of HIV, syphilis and hepatitis B [36]. Furthermore, integration of syphilis and hepatitis B testing in nationally representative surveys for HIV and other health conditions would further strengthen the availability of data needed for validation of EMTCT of syphilis and HBV, as well as monitoring disease burden to inform national priorities with regard to prevention, screening and treatment for syphilis and hepatitis B for pregnant women [37].

Progress towards HIV EMTCT is highly variable across these high‐burden countries and shows regional patterns, with the southern African countries of Botswana, Eswatini, Lesotho, South Africa and Namibia all estimated to have ≤5% MTCT rates. Persistent gaps in HIV PMTCT service delivery remain in many high‐burden countries. These include limited or delayed uptake of HIV testing and ART, treatment interruptions, and incident HIV infection during pregnancy or breastfeeding. Uptake of HIV testing and treatment among pregnant and breastfeeding women is particularly low in areas where many women do not receive skilled ANC and/or do not deliver at a health facility [38]. PEPFAR programme results indicate 100% HIV testing coverage in ANC and 99% ART coverage among pregnant women in PEPFAR‐supported sites in 2024 [39], yet population‐level data show HIV testing and treatment gaps among pregnant women due to access to health facilities with comprehensive PMTCT services. Improving accessibility of services, conducting community outreach and providing peer support can improve PMTCT uptake and retention [40, 41]. Treatment interruptions during pregnancy and breastfeeding are often related to challenges reaching or receiving timely care at a health facility and are more common among women who started ART during pregnancy and among adolescent and young mothers [42, 43, 44]. In high HIV prevalence and incidence countries in east and southern Africa, where nearly one‐third of infant HIV infections are attributable to women acquiring HIV during pregnancy or the postnatal period, improving access to and uptake of HIV prevention services in pregnancy and post‐partum, including pre‐exposure prophylaxis, is a particularly high‐impact way to reduce HIV MTCT [45, 46, 47].

For syphilis, challenges with maternal testing and treatment during ANC hinder progress towards and monitoring of elimination goals for CS. Indeed, four of the five countries with maternal syphilis testing coverage of <50% did not report congenital case rates. Laboratory testing can incur a cost for pregnant women and may require travel for serum specimen collection, leading to a lack of testing or delays in the return of syphilis results and treatment [48]. WHO‐recommended use of quality‐assured rapid dual HIV/syphilis tests as the first HIV test during ANC has accelerated syphilis screening and prompt treatment, facilitated by national‐level procurement and implementation through international donor support [49, 50, 51, 52, 53]. Quality, procurement, supply shortages and administration of benzathine penicillin, the only recommended treatment for pregnant women testing positive for syphilis, remain ongoing challenges to ensuring timely and appropriate maternal treatment for prevention of CS [54, 55, 56, 57]. Challenges in appropriate diagnosis and costs of infant hospitalization and intravenous therapy with penicillin G for CS can be barriers to appropriate infant treatment [52, 57]. Finally, CS case definitions vary at the country level, challenging national eligibility for validation of EMTCT or PTE, which requires the use of the WHO case definition [1].

For hepatitis B, the majority of countries assessed have not introduced the HepB‐BD in their national immunization schedule, and data are lacking on uptake of hepatitis B testing and treatment among pregnant women. Several barriers have been identified among patients, providers, surveillance and healthcare systems that hinder prevention, screening, treatment and monitoring for hepatitis B among pregnant women and their infants [58, 59, 60]. Cost barriers hinder scale up of HepB‐BD introduction and implementation of nationwide testing and treatment for hepatitis B. In most countries, women have to pay out of pocket for those services and require travel to specialized health facilities to seek care and treatment for hepatitis B [58, 60]. Provider barriers include a lack of adequate knowledge on the importance of timely HepB‐BD coverage [58, 61], while healthcare system barriers included lack of commodities (test kits, vaccines, antiviral treatment), and limited facility births at primary healthcare centres [36, 62, 63]. Limited awareness of hepatitis B burden in pregnancy and prevention of vertical transmission [58, 62] can be further challenged by the lack of consistently reported data on antenatal coverage of HBsAg testing and antiviral treatment for hepatitis B during pregnancy. The 2024 WHO hepatitis B treatment guidelines remove barriers related to access to and cost of viral load testing since all pregnant women testing HBsAg positive are eligible for treatment with tenofovir disoproxil fumarate [2, 10]. Decentralization of hepatitis B treatment to the primary healthcare level, similar to HIV, would be necessary to address access barriers faced by pregnant women.

Implementation of universal, cost‐effective [64, 65, 66, 67, 68] PMTCT interventions for these three infections at patient‐care levels requires scale up, staff training [69, 70] and maintenance of commodity supply. Programme coordination across antenatal, newborn, paediatric and immunization platforms is necessary to ensure prevention of infant hepatitis B through maternal testing, treatment, birth dose and childhood hepatitis B vaccination. Integration of services for PMTCT of HIV, HBV and syphilis provides opportunities to advance triple EMCT through integrated service delivery within ANC, and it requires collaboration across governmental, community‐based, civil society and healthcare service providers [1, 37, 38].

Limitations regarding this analysis are primarily related to data availability, variability and quality. First, essential data on EMTCT service delivery indicators were missing for several countries or reflected data collected many years prior. Second, service delivery for HIV treatment coverage exceeded 100% in some countries due to the processes for population‐based treatment estimates, and population‐based HIV testing and treatment coverage cannot be directly compared to the ANC facility‐based syphilis testing and treatment coverage indicators. Third, country‐reported CS rates may not consistently reflect the WHO case definition, which includes stillbirths and asymptomatic infants born to women with inadequately treated syphilis. Fourth, country‐reported CS rates do not account for the background prevalence of syphilis among pregnant women who did not attend ANC and thus did not have access to testing and treatment. Fifth, not all countries reported timely HepB‐BD coverage, and none of the countries report screening and treatment coverage for hepatitis B among pregnant women. Finally, most of the hepatitis B prevalence estimates in children were based on modelled estimates with very few nationally representative serosurveys.

## 5 CONCLUSIONS

While many countries have made progress, challenges in reaching EMTCT of HIV remain in these countries, and service delivery coverage for EMTCT of syphilis and hepatitis B lags behind that of HIV. ANC and HIV PMTCT platforms offer optimal clinical settings to incorporate syphilis and hepatitis B services to prevent MTCT. Commitment to EMTCT service delivery is evident within national policies and from policymakers within these countries [70]. Limited supplies of diagnosis, treatment and vaccine commodities and out‐of‐pocket costs for pregnant women result in missed opportunities for maternal syphilis and hepatitis B testing and treatment, and hepatitis B vaccination alongside efforts for HIV PMTCT [71]. In the changing funding landscape, ongoing investment is needed for the advancement of triple EMTCT of HIV, syphilis and hepatitis B through integrated service delivery covering all three infections for pregnant women and common data systems that track EMTCT indicators [1, 37, 72].

## COMPETING INTERESTS

The authors have no conflicts to declare.

## AUTHOR CONTRIBUTIONS

MT contributed to the supervision, conceptualization, methodology, analysis, writing of original draft and review and revision of final manuscript draft. RT, MM, and RM contributed to the conceptualization, methodology, analysis, writing and revision of final manuscript draft.

## FUNDING

Authors are U.S. government employees. No additional funding was received to support this review.

## DISCLAIMER

The views expressed in this manuscript are those of the authors and do not necessarily represent the official position of the U.S. Centers for Disease Control and Prevention.

## ETHICS APPROVAL STATEMENT

This activity was reviewed by CDC, deemed not research, and was conducted consistent with applicable federal law and CDC policy.

## Supporting information




**Table S1**: Data sources for evaluation of progress towards elimination of mother‐to‐child transmission of HIV, syphilis and hepatitis B

## Data Availability

Publicly available data were used for this review and are presented within the manuscript.
