# Supplementary material for: Progress towards the elimination of vertical transmission of HIV, syphilis and hepatitis B in 21 high‐burden countries
Source: J Int AIDS Soc. 2026 Mar 26;29(4):e70091. doi: 10.1002/jia2.70091 (PMC13140317; doi:10.1002/jia2.70091)
Supplement: Supplementary file 1 — Table S1: Data sources for evaluation of progress towards elimination of mother‐to‐child transmission of HIV, syphilis and hepatitis B [file JIA2-29-e70091-s001.docx]

**Supplemental Table 1: Data sources for evaluation of progress towards elimination of mother-to-child transmission of HIV, syphilis and hepatitis B**

|  | **Data sources** | **Indicators** |
| --- | --- | --- |
| Antenatal care | UNICEF [11] | 1. Antenatal care coverage of at least one visit (ANC1) 2. Facility delivery coverage (births with skilled birth attendant) |
| HIV | UNAIDS (aidsinfo.unaids.org) [12-15] | 1. HIV testing coverage in ANC/pregnant women 2. ART coverage in pregnant women (population) 3. HIV prevalence in women ages 15-49 4. MTCT rate (population) |
| Syphilis | WHO and UNAIDS Global AIDS Monitoring (Located in the Global Health Observatory) [12,16] | Annually reported GAM syphilis indicators  (prior 12 months)  (1) Syphilis testing coverage among pregnant women  (2) Syphilis test positivity among pregnant women  (3) Syphilis treatment coverage among pregnant women diagnosed with syphilis  (4) Number/rate of congenital syphilis cases  (5) Syphilis test type used |
| Hepatitis B | - WHO and UNICEF estimated vaccination coverage [17] - Population-Based HIV Impact Assessment surveys (PHIA) [18-23] - Other nationally representative surveys and modeled estimates [10, 24-30] | 1. Hepatitis B birth dose vaccination coverage 2. Hepatitis B infant immunization rates 3. Hepatitis B seroprevalence in children 4. Hepatitis B seroprevalence in women of childbearing age or pregnant women |
| National Policies | WHO and UNAIDS [13,31-32] | 1. National EMTCT policies and plans 2. National policies for screening of hepatitis B in pregnancy |
